# Patient-derived organoids as a predictive biomarker for treatment response in cancer patients

**DOI:** 10.1038/s41698-021-00168-1

**Published:** 2021-04-12

**Authors:** G. Emerens Wensink, Sjoerd G. Elias, Jasper Mullenders, Miriam Koopman, Sylvia F. Boj, Onno W. Kranenburg, Jeanine M. L. Roodhart

**Affiliations:** 1 Department of Medical Oncology, University Medical Center Utrecht, Utrecht University, Utrecht, the Netherlands; 2 Department of Epidemiology, Julius Center for Health Sciences and Primary Care, University Medical Center Utrecht, Utrecht University, Utrecht, the Netherlands; 3 Foundation Hubrecht Organoid Technology (HUB), Utrecht, the Netherlands; 4 Department of Surgical Oncology, University Medical Center Utrecht, Utrecht University, Utrecht, the Netherlands; 5 Utrecht Platform for Organoid Technology, University Medical Center Utrecht, Utrecht University, Utrecht, the Netherlands

**Keywords:** Cancer therapy, Predictive markers, Translational research

## Abstract

Effective predictive biomarkers are needed to enable personalized medicine and increase treatment efficacy and survival for cancer patients, thereby reducing toxic side effects and treatment costs. Patient-derived organoids (PDOs) enable individualized tumour response testing. Since 2018, 17 publications have examined PDOs as a potential predictive biomarker in the treatment of cancer patients. We review and provide a pooled analysis of the results regarding the use of PDOs in individualized tumour response testing, focusing on evidence for analytical validity, clinical validity and clinical utility. We identify future perspectives to accelerate the implementation of PDOs as a predictive biomarker in the treatment of cancer patients.

## Introduction

Despite advances in the treatment of cancer patients, the burden of cancer deaths remains high, with 9,5 million cancer deaths reported worldwide in 2018^1^. A key limitation in cancer treatment is the lack of valid predictive biomarkers, which reduces the efficacy of treatments^2^. Oncologists are largely unable to predict treatment response for individual patients, resulting in patients receiving ineffective treatment with unnecessary exposure to toxic side effects and high treatment costs. Effective predictive biomarkers are needed to enable personalized medicine and increase survival for cancer patients. Personalized medicine strategies include protein-, RNA-based and genome-based stratification, though in oncology, precision medicine has been largely based on genomic biomarkers^3^. However, less than half of patients are eligible for genetically matched treatment^4,5^ and for the majority of anticancer agents no genetic markers are available.

**Fig. 1:**
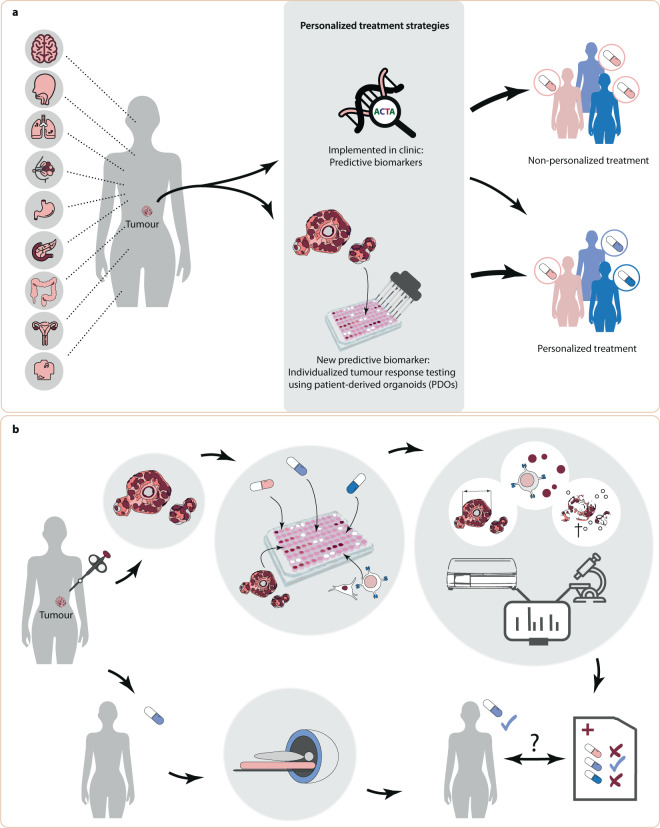
PDO-based individualized tumour response testing as a predictive biomarker. **a** Illustrates the tumour types for which patient-derived organoids (PDOs) have been tested for clinical validity (listed in full in Table 1). Personalized treatment strategies currently implemented in oncology treatment largely comprise of genomic biomarkers. However, this only results in a personalized treatment strategy for a minority of patients. Individualized tumour response testing using PDOs is a new biomarker which may be used in personalized treatment and increases access to personalized treatment. **b** For individualized tumour response testing, tissue from a patient’s tumour is obtained to culture organoids, perform drug screens and various read-outs can be obtained to define PDO drug screen response (including organoid size, viability and co-culture cytokine measurements). A predictive biomarker test is developed using the PDO drug screen results and clinical response seen in patients.

A promising predictive biomarker is individualized tumour response testing using patient-derived organoids (PDOs), in which anticancer agents are screened ex vivo on PDOs to predict clinical response (Fig. 1). PDOs have been developed for a variety of tumours and are stem-cell derived, three-dimensional self-organizing structures comprised of epithelial cells, mimicking its corresponding tumour^6,7^. PDOs represent a superior preclinical model system compared to previous models through their inherent heterogeneity, long-term stability, applicability for high-throughput screens and enhanced capacity to capture tumour characteristics^8–10^. In 2018 it was first demonstrated that PDOs may predict treatment response in cancer patients^11^.

In order to perform individualized tumour response testing with PDOs, tissue is obtained from a patient’s tumour to culture PDOs and perform drug screens (Fig. 1). Treatment efficacy is measured by analysing potential end points which are correlated with treatment sensitivity, including organoid size, viability and co-culture cytokine measurements. Finally, drug screen results and clinical response data are combined to create predictive biomarker tests which are capable of predicting treatment response in patients for a given treatment. Three qualities must be fulfilled for PDOs to function as an effective biomarker: analytic validity, clinical validity and clinical utility^12^. Tests to derive the predictive biomarker should be accurate, reproducible and robust (analytically valid) and results must correlate with clinical end points (clinically valid)^12^. The use of the predictive biomarker should result in improved patient outcome (clinical utility) compared to standard of care treatment, in a cost-effective manner.

In this systematic review, we identified 17 oncological studies which report data regarding PDO-based drug screen results and their predictive value or association with the patient’s response to treatment in the clinic (Table 1, search strategy is described in Supplementary Table 1). We evaluate the analytical validity by reviewing different drug screen methods used. Next, we investigate the clinical validity by evaluating if clinical studies demonstrate a correlation between PDO-based drug screen results and clinical treatment response in patients and assess if this is impacted by intra-patient heterogeneity. We explore aspects related to the clinical utility and the feasibility of using PDOs in the clinic, including establishment rate and time needed to obtain PDO drug screen results. Lastly, we offer perspectives for future research.

**Table 1:** Study characteristics.

Publication (author, year, study name)	Study design	Tumour type & stage	Drug screen cohort		Clinical response association cohort		Treatment
			Patients	PDOs	Patients	PDOs	
Ooft, 2019^21^ TUMOROID	Prospective cohort, observational	mCRC	29	35	29	35	FOLFIRI (>1st line); Irinotecan (>1st line); FOLFOX (mixed lines)
Chalabi, 2020^22^ NICHE	Prospective cohort (within phase II trial), observational	CRC (Stage III)	11	12	11	12	Nivolumab + ipilimumab (neoadjuvant)
Ganesh, 2019^29^	Observational cohort	RC (non-metastatic & metastatic)	14	23	9	17	5-FU & FOLFOX; Radiation
Yao, 2020^26^ CinClare	Prospective, observational (within phase III trial)	LARC	80	80	80	80	Chemoradiation (capecitabine versus CAPIRI; neoadjuvant)
Narasimhan, 2020^28^ APOLLO	Prospective, offers assay-guided treatment to treatment refractory patients	mCRC (peritoneal)	15	17	9	9	FOLFOX, FOLFIRI, regorafenib, vandetanib, gemcitabine
Vlachogiannis, 2018^11^	Prospective, observational, using PDOs from 4 prospective phase I/II trials	mCRC, mGC, mGOC	15	19	15	19^a^	TAS-102, Cetuximab, Regorafenib (CRC); Paclitaxel (GC); 5-FU + cisplatin (GOC)
Steele, 2019^31^	Observational cohort	GC (non-metastatic & metastatic)	6	6	2	2	EOX
Tiriac, 2018^23^	Observational cohort	Pancreatic cancer (Stage II–IV)	57	66	9	12	5-FU; Gemcitabine + nab-paclitaxel; 5-FU + SN-38 + gemcitabine; 5-FU + SN-38 + oxaliplatin; 5-FU + oxaliplatin; 5-FU + gemcitabine
Sharick, 2020^17^	Observational cohort	Pancreatic cancer (non-metastatic); Breast cancer (not specified)	24	24	10	10	Gemcitabine + 5-FU, oxaliplatin + 5-FU, 5-FU or FOLFIRINOX (pancreatic cancer). AC-T (breast cancer)
Li, 2018^27^	Observational cohort	Oesophageal cancer (non-metastatic)	8	8	5	5	ECX, ECF, CF
Driehuis, 2019^34^	Observational cohort	HNSCC (non-metastatic)	14	14	7	7	Radiation (postoperative with curative intent, primary and adjuvant)
Sachs, 2018^14^	Observational cohort	Breast cancer (metastatic)	NR	12	2	2	Tamoxifen
Phan, 2019^18^	Observational cohort	Ovarian carcinoma (Stage IV)	4	4	2	2	Carboplatin
De Witte, 2020^32^	Observational cohort	Ovarian carcinoma (non-metastatic & metastatic)^b^	23	36	5	7	Carboplatin + paclitaxel.
Votanopoulos, 2019^19^	Observational cohort	Melanoma (Stage III–IV)	7	9	5	7	Pembrolizumab, nivolumab, ipilimumab, dabrefinib/trametinib
Mazzocchi, 2018^20^	Observational cohort	Mesothelioma (metastatic)	2	2	2	2	Cisplatin + pemetrexed.
Jacob, 2020^30^	Observational cohort	Glioblastoma (WHO grade IV)	7	8	5	6	Radiation + temozolomide.

A description of the types of tumours (and stages), cohort size and examined treatments (lines) used for the comparison between ex vivo PDO drug screen results and clinical treatment response are reported. The number of patients and PDOs in the study in which drug screens were performed are reported, as well as the number of patients and PDOs for which an association was made with the clinical treatment response.

*5-FU* 5-flourouracil, *AC-T* doxorubicin + cyclophosphamide + paclitaxel, *CAPIRI* capecitabine + irinotecan, *CF* cisplatin + 5-FU, *ECF* epirubicin + cisplatin + 5-FU, *ECX* epirubicin + cisplatin + capecitabine, *EOX* epirubicin + oxaliplatin + 5-FU, *FOLFIRI* 5-FU + irinotecan, *FOLFIRINOX* 5-FU + oxaliplatin + irinotecan, *FOLFOX* 5-FU + oxaliplatin, *HNSCC* head and neck squamous cell carcinoma, *LARC* locally advanced rectal cancer, *mCRC* metastatic colorectal cancer, *mGC* metastatic gastric cancer, *mGOC* metastatic gastroesophageal cancer, *mRC* metastatic rectal cancer, *NR* not reported, *PDO* patient-derived organoid, *RC* rectal cancer, *SN-38* irinotecan, *TAS-102* trifluridine/tipiracil, *WHO* World Health Organization.

^a^The authors report diagnostic results for 21 organoids (2 organoids were tested for >1 treatment line), so that the clinical cohort consists of 19 unique organoids.

^b^The clinical comparison cohort (*n* = 5) comprised of high-grade serous ovarian cancer patients who underwent interval debulking surgery.

## Analytic validity: PDO-based drug screen experimental set-up

Prior to performing drug screens, it is essential to perform quality control to verify that the PDOs have been cultured in adequate growth medium to avoid selection bias during establishment and represent the patient’s tumour without overgrowth of normal tissue^13^. The 17 studies in this review used varying medium compositions. Depending on the tumour type, growth factor requirements may vary (e.g. neuregulin in breast cancer organoids^14^ and β-estradiol in ovarium cancer organoids^15^), however, even within the same tumour type medium compositions and culturing techniques differ. Serum-free growth factors are increasingly becoming available, which allows establishment of organoids in serum-free medium, without undefined differentiation-inducing components^16^. Of note, three studies used serum-based media^17–19^, while one study did not specify which medium was used to establish organoids^20^. All studies used at least one of the following quality control assays to verify that PDOs reflect the original tumour: histopathology morphological assessment, DNA and/or RNA sequencing, niche-dependency assays and/or engraftment of organoids in mice (summarized in Supplementary Table 2). In four studies, genomic analysis included criteria for which PDOs could be excluded from analysis due to poor quality^14,21–23^. Of note, in several studies quality control was performed on a subset of PDOs, not the whole cohort. A recent protocol for establishing PDOs for drug screening suggests to perform genetic analysis of PDOs and the original tumour tissue to assess that the PDOs are representative and match with the tumour^13^.

The experimental set-up used for PDO-based individualized tumour response testing differed for each study (Supplementary Table 2). PDO drug screens were performed using PDOs embedded in a matrix, in suspension and in a co-culture model. The duration of exposure to drugs varied from 2 to 24 days. Different end point read-outs were chosen: often cell viability in a luminescence assay (11/17 studies), but also including immunofluorescence with a dead/alive staining, and quantification of interferon-gamma (IFN-ƴ) in CD8+ T-cells. The TUMOROID trial included a baseline viability measurement in its drug screen set-up, which allowed the determination of growth rate inhibition metrics (GR)^21^, an approach which takes into account the proliferation rate, a known source of variance in drug screens^24^. Sharick et al. used optical metabolic imaging (OMI) to measure the metabolic state of single cells within PDOs relative to the average of control cells, which is unique in capturing metabolic heterogeneity during treatment in addition to treatment effect size^17^.

When specified how in vitro response was defined, the most frequently used index test was area under the drug response curve (AUC; in seven studies) rather than other drug response curve (DRC) parameters (listed in Table 2 as the index test). Not all studies provided a definition for in vitro response. The parameters which are most informative in predicting patient response may be drug or disease specific^24^. The AUC of a DRC, which combines the potency and efficacy of a drug, is a robust parameter when aiming to compare one agent across multiple tissue lines exposed to the same concentration range and may be more accurate than IC_50_ (50% inhibitory concentration)^24,25^. Lastly, for combination treatment two approaches were used to define in vitro response: analysing each agent separately for a combined response classification^19,21,23,26,27^ or analysing the response to combination treatment directly in vitro^17,20,21,26–32^. The CinClare trial and de Witte et al. report evidence of synergism for combination treatment^26,32^. The TUMOROID trial reported a significant difference in the drug screen results for irinotecan double treatment between PDOs with progressive disease (PD) versus partial response/stable disease (PR/SD; *p* = 0.0260), whereas there was no significant difference in the individual drug parameter drug screen results^21^. Analysing combination drug screen results, rather than each drug separately, may more accurately discriminate the clinical response in patients.

**Table 2:** Clinical association with drug screen results.

Tumour type [Study]	Treatment	*n*	PDOs	Events	Index test (PDO)	Reference test (clinic)	AUROC [95% CI]	Sens/Spec/PPV/NPV	Group values or correlation results
mCRC [Ooft^21^]	FOLFIRI (>1st line)	12	12	6	GR50 (single treatments)	RECIST response of target lesion^1^	SN-38: 0.83 [0.59–1.08]; 5-FU: 0.80 [0.53–1.08]		
					AUC (single treatments)	RECIST response of target lesion^1^	SN-38: 0.75 [0.44–1.06]; 5-FU: 0.80 [0.53–1.08]		
					Pan-matrix GR score	RECIST response of target lesion^1^	0.83 [0.53–1.13]	Sens: 1.00; Spec: 0.83; PPV: 0.86; NPV**:** 1.00	
					GR score-based FOLFIRI classifier	RECIST response of target lesion^1^	0.89 [0.67–1.11]	Sens: 1.00; Spec: 0.83; PPV**:** 0.86; NPV: 1.00	GR score PD 0.73 [0.13–1.43]; GR score PR/SD −1.88 [−1.61 to −2.13; *p* = 0.0260.
					GR score-based FOLFIRI classifier	PFS			Median PFS of 50% most sensitive PDOs 169 days; Median PFS of 50% most resistant PDOs 58 days; log rank *p* = 0.0278.
					GR score-based FOLFIRI classifier	RECIST response of target lesion^1^ (on irinotecan monotherapy cohort)	0.84 [0.54–1.14]	Sens: 1.00; Spec: 0.83; PPV: 0.80; NPV**:** 1.00	Correct classification 83.3% patients (*p* = 0.0017).
mCRC [Ooft^21^]	Irinotecan (>1st line)	10	10	5	GR50	RECIST response of target lesion^1^	0.96 [0.84– 1.11]		Median GR50 PD 0.0053 [0.0047–0.0058]; Median GR50 SD 0.0023 [0.0019–0.0027]; *p* = 0.0159.
					AUC	RECIST response of target lesion^1^	(reference in figure)		Median AUC PD 0.70 [0.67– 0.72]; Median AUC SD 0.54 [0.52–0.57]; *p* = 0.0079.
					GR score-based irinotecan classifier	RECIST response of target lesion^1^			Correct classification 80% patients (*p* = 0.0061).
					GR score at 3.2 nM SN-38	RECIST response of target lesion^1^	0.96 [0.84– 1.11]	Sens: 1.00; Spec: 0.80; PPV**:** 0,83; NPV: 1.00	Median GR score PD 0.70 [0.63–0.77; Median GR score SD 0.33 [0.26–0.40]; Mann Whitney test *p* = 0.0159.
mCRC [Ooft^21^]	FOLFOX (mixed lines)	10	16	4	GR50 (single treatments)	RECIST response of target lesion^1^	Oxal.: 0.69 [0.40–0.99]; 5-FU: 0.55 [0.17–0.94]		GR50 5-FU PD 5.17 [3.22-8.31]; GR50 5-FU PR/SD 3.91 [3.00–5.36]; *p* = 0.7105. GR50 oxaliplatin PD 104.2 [89.2–123.2]; GR50 oxaliplatin PR/SD 71.3 [51.6–97.1], *p* = 0.3301.
					AUC (single treatments)	RECIST response of target lesion^1^	Oxal.: 0.69 [0.40–0.99]; 5-FU: 0.56 [0.22–0.89]		AUC 5-FU PD 0.81 [0.78–0.83]; AUC 5-FU PR/SD 0.77 [0.74–0.81]; *p* = 0.9399. AUC oxaliplatin PD 0.96 [0.95–0.96]; AUC oxaliplatin PR/SD 0.89 [0.84–0.94]; *p* = 0.3301.
					GR score-based (diagonal DRC combination)	RECIST response of target lesion^1^	0.56 [0.11–1.01]		GR score PD 1.52 [0.84-2.24]; GR score PR/SD 1.71 [1.38 - 2.04]; not significant.
					Pan-matrix GR score	RECIST response of target lesion^1^	0.59 [0.19–0.98]	Sens: 0.89^; Spec: 0.50^; PPV**:** 0.80^; NPV: 0.67^	
CRC [Chalabi^22^]	Immune checkpoint inhibitors (neoadjuvant)	11	12	6^≠^	IFN-ƴ (CD-8+ T-cells)	Pathological: MPR(≤10% RVT). PR (≤50% RVT) or MPR/PR are considered response		Sens: 0.50^; Spec: 1.00^; PPV: 1.00^; NPV: 0.67^	
RC [Ganesh^29^]	5-FU & FOLFOX	7	14	7	AUC	PFS			Spearman *r* = −0.86, *p* = 0.024 for AUC & PFS.
	Radiation	7	11	3	AUC	Endoscopic response to radiation (% tumour decrease)			% AUC matched clinical endoscopic clinical response to radiation.
LARC [Yao^26^]	Chemoradiation (cap. or CAPIRI; neoadjuvant)	80	80	43	Organoid size decrease cut-off (sensitive if ≥1/3 sensitive)	Pathological: TRG 0–1 (good); TRG 2–3 (poor)	0.88 [0.76–0.99]	Sens**:** 0.92 ^2^; Spec: 0.79 ^2^; PPV**:** 0.79; NPV**:** 0.92	
	(Radiation only)				Organoid size decrease cut-off (sensitive if ≥1/3 sensitive)	Pathological: TRG 0–1 (good); TRG 2–3 (poor)		Sens: 0.41; Spec: 0.98; PPV: 0.94; NPV: 0.66	
	(5-FU only)				Organoid size decrease cut-off (sensitive if ≥1/3 sensitive)	Pathological: TRG 0–1 (good); TRG 2–3 (poor)		Sens: 0.59; Spec: 0.88; PPV: 0.81; NPV: 0.72	
	(Irinotecan only)				Organoid size decrease cut-off (sensitive if ≥1/3 sensitive)	Pathological: TRG 0–1 (good); TRG 2–3 (poor)		Sens: 0.78; Spec: 0.79; PPV: 0.78; NPV: 0.79	
mCRC [Narasimhan^28^]	FOLFOX	5	5	3	AUC	PD vs. PR/SD			AUC in PR/SD vs. PD, no statistically significant difference.
mGIC [Vlachogiannis^11^]	Combined	15	19^*^	14^≠^	Not specified	Not specified		Sens: 1.00^; Spec: 0.93^; PPV: 0.88^; NPV: 1.00^	
Gastric cancer [Steele^31^]	EOX	2	2	1	% cell viability decrease (flow cytometry)	Pathological: grade (% residual tumour)		Sens: 1.00; Spec: 1.00; PPV: 1.00; NPV**:** 1.00	
Pancreatic cancer [Tiriac^23^]	Combined	9	12	3	AUC (tertiles of sensitive/intermediate/resistant; separately/drug)	PFS (relative to expected median)		Sens: 1.00; Spec: 0.67; PPV: 0.86; NPV: 1.00	Patients with PFS > median (*n* = 6): 5/6 PDOs had >1 sensitive drug and no resistant drugs in vitro.
Pancreatic cancer [Sharick^17^]	Combined (adjuvant)	7	7	3	OMI index (metabolic state)	RFS (>12 months)		Sens: 1.00; Spec: 1.00; PPV: 1.00; NPV: 1.00	Median OMI index in RFS > 12 months 2.2 [1.2–3.2]; Median OMI index in RFS < 12 months 0.4 [0.2–0.5]. Decreased heterogeneity of treatments/controls in RFS > 12 months vs. RFS < 12 months.
Oesophageal cancer [Li^27^]	Combined	5	5	5	AUC (monotherapy and combination)	Pathological: (resistant: TRG 4–5)		Sens: – Spec: 0.60; PPV: 0.00; NPV: 1.00	
	ECX	3	3	3	AUC (monotherapy and combination)	Pathological: (resistant: TRG 4–5)		Sens: –; Spec: 0.67; PPV: 0.00; NPV: 1.00	
	ECF	1	1	1	AUC (monotherapy and combination)	Pathological: (resistant: TRG 4–5)		Sens: – Spec: 1.00; PPV: – NPV: 1.00	
	CF	1	1	1	AUC (monotherapy and combination)	Pathological: (resistant: TRG 4–5)		Sens: – Spec: 0.00; PPV: 0.00; NPV: –	
HNSCC [Driehuis^34^]	Radiotherapy (curative intent)	7	7	3	AUC	Relapse after radiation		Sens: 0.75; Spec: 1.00; PPV: 1.00; NPV: 0.75	
Ovarian cancer [Phan^18^]	Carboplatin	2	2	2	Decrease in viability	Not specified		Sens: -; Spec: 1.00; PPV: –; NPV: 1.00	Descriptive results (*n* = 2).
Ovarian cancer [De Witte. 2020^32^]	Carboplatin + paclitaxel	5	7	4^≠^	AUC	(1) Pathological [CRS = 1 vs. CRS = 2]			AUC higher in CRS = 1 vs. CRS = 2; Wilcoxon signed-rank test *p* < 0.001.
					AUC	(2) CA-125 normalization (<35 kU/L during 1^o^ treatment)			AUC higher in non-CA-125 normalization vs. normalization; *p* = 0.0004.
					AUC	(3) RECIST response (SD vs. PR)			AUC higher in RECIST SD versus PR; *p* = 0.0092.
					AUC	(4) 6-month PFS			AUC did not correlate with 6-month PFS; *p* = 0.9663.
					AUC	(5) OS			AUC did correlate with OS (<17-months); Wilcoxon signed-rank test corrected for multiple testing *p* = 0.0016.
Melanoma [Votanopoulos^19^]	Immune checkpoint inhibitors	5	7	5	Decrease in viability	Not specified		Sens**: –**; Spec: 0.60; PPV**:** 0.00; NPV**:** 1.00	
	Dabrefinib/trametinib	1	2	1^≠^	Decrease in viability	Not specified		Sens**:** 1.00^; Spec: 1.00^; PPV**:** 1.00^; NPV**:** 1.00^	
Mesothelioma [Mazzocchi^20^]	Cisplatin + pemetrexed	2	2	1	Decrease in viability	Not specified		Sens: 1.00; Spec: 1.00; PPV: 1.00; NPV**:** 1.00	
Glioblastoma [Jacob^30^]	Radiation + temozolomide	5**	6	3	Decrease in viability	Not specified		Sens: 1.00; Spec: 1.00; PPV**:** 1.00; NPV**:** 1.00	

Results concerning clinical validity results for PDO drug screens and clinical response are displayed, along with the publication, tumour type & stage, parameters compared, treatment, number of patients, PDOs and events per treatment. Where applicable, results were digitized from graphs using WebPlotDigitizer software. ^Sensitivity/specificity/PPV/NPV are calculated using patient-level values, except for ref. ^21^: outcomes are reported for 2 PDOs per patient for three patients with PDOs before & after treatment^22^; includes 2 PDOs for 1 patient with synchronous dMMR and pMMR tumours^11^; results for PDO-level (not available for patient-level). *The authors report diagnostic results for 21 organoids (2 organoids were tested for >1 treatment line), so that the clinical cohort consists of 19 unique organoids, of which regorafenib in vitro tests were performed using xenograft models (not a PDO drug screen). ** Drug screen performed in 7 patients, but clinical response reported for 5 patients. ^1^indicates response was measured after three cycles of treatment; ^2^the reported diagnostic values differ from the article due to alternative designation of true positive and true negative values, ^≠^Only reported for PDOs, not patient-level^11^; patient had 2 PDOs with mixed clinical response^19,32^ or patient had synchronous tumour (responders & non-responder) with 2 PDOs^22^.

*1*^*o*^ primary treatment, *5-FU* 5-flourouracil, *AUC* area under the curve, *AUROC* area under the receiver operator curve, *CI* confidence interval, *capec*. capecitabine, *CAPIRI* capecitabine + irinotecan, *CAPOX* capecitabine + oxaliplatin, *CF* cisplatin + 5-FU, *CR* complete response, *CRS* chemotherapy response score, *dMMR* deficient mismatch repair, *DRC* drug response curve, *ECF* epirubicin + cisplatin + 5-FU, *ECX* epirubicin + cisplatin + capecitabine, *EOX* epirubicin + oxaliplatin + 5-FU, *FOLFIRI* 5-FU + irinotecan, *FOLFOX* 5-FU + oxaliplatin, *GR* growth rate inhibition metrics, *GR*_*50*_ concentration at which GR is 50%, *HNSCC* head and neck squamous cell carcinoma, *IFN-ƴ* interferon gamma, *LARC* locally advanced rectal cancer, *mCRC* metastatic colorectal cancer, *mGIC* metastatic gastrointestinal cancer, *MPR* major pathological response, *NPV* negative predictive value, *OMI* optical metabolic imaging, *OS* overall survival, *Oxal.* oxaliplatin, *PD* progressive disease, *PDO* patient-derived organoid, *PFS* progression-free survival, *pMMR* proficient mismatch repair, *PPV* positive predictive value, *PR* partial response, *RC* rectal cancer, *RECIST* response evaluation criteria in solid tumours, *RFS* recurrence-free survival, *RVT* residual viable tumour, *SD* stable disease, *sens.* sensitivity, *SN-38* irinotecan, *spec.* specificity, *TRG* tumour regression grade score.

## Clinical validity: correlation of PDO drug screen sensitivity with clinical response

The 17 studies in this review assessed the clinical validity of PDOs as a predictive biomarker for treatment response in the clinic. The studies were heterogeneous, varying in study design, patient population and treatments (Table 1). All studies were observational, with the exception of the APOLLO trial which was the first study to offer patients assay-guided treatment^28^. The results encompassed a variety of tumour types and stages of disease. Colorectal cancer (CRC) studies were the most frequent among the publications (5/17) and also the largest in patient cohort size^21,22,26,28,29^. Many studies (7/17) derived PDOs from patients with metastatic disease^11,14,18,20,21,28,30^. Lastly, the treatments examined included systemic chemotherapy, targeted therapy, (chemo)radiation and immunotherapy.

In general, the patient cohorts for which ex vivo drug response results and clinical response are available were small, varying from 2 to 80 patients per study, with a median of 7 patients per study and a median of 3 patients per type of treatment per study (Tables 1 and 2). An exception is the Phase 3 CinClare trial, which examined PDO drug response in 80 locally advanced rectal cancer (LARC) patients receiving neoadjuvant chemoradiation, randomized for capecitabine versus capecitabine with irinotecan (CAPIRI)^26^.

**Fig. 2:**
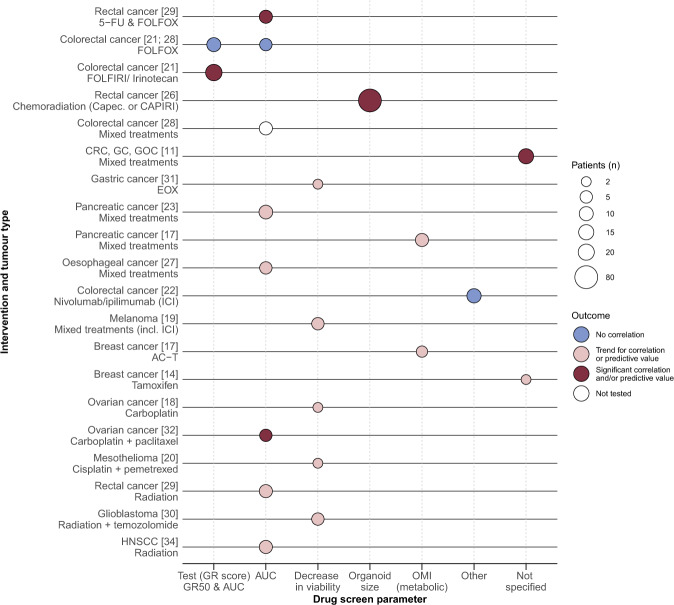
Evidence landscape of PDO drug screen parameters and clinical response. Illustrates the clinical validity results for PDOs as a predictive biomarker for treatment response (dark red: significant correlation and/or predictive value found, pink: trend for correlation or predictive value found, blue: no correlation and white: not tested), with the size of the circle representing the patient cohort size, specified per treatment and tumour type (*y*-axis) and ex vivo drug response parameter (*x*-axis). *Abbreviations:* 5-FU 5-fluorouracil, AC-T doxorubicin + cyclophosphamide + paclitaxel, AUC area under the curve, Capec*.* capecitabine, CAPIRI capecitabine + irinotecan, CRC colorectal cancer, EOX epirubicin + oxaliplatin + 5-FU, FOLFIRI 5-FU + irinotecan, FOLFOX 5-FU + oxaliplatin, GC gastric cancer, GOC gastroesophageal cancer, GR growth rate inhibition metrics, GR50 value with 50% viable GR, HNSCC head and neck squamous cell carcinoma, ICI immune checkpoint inhibitors, OMI optical metabolic imaging, PDO patient-derived organoid.

The results regarding the correlation of PDO-based drug screen results and clinical response per study are described per tumour type and treatment type below (Table 2 and Supplementary Table 3). We summarized the clinical validity results for all studies into an evidence landscape figure (Fig. 2)^33^. Five of the 17 studies reported a statistically significant correlation and/or predictive value for PDO-based drug screen results and clinical response for a given treatment^11,21,26,29,32^. A trend for a correlation or predictive value was seen in 11 studies for a given treatment^14,17–20,23,27,29–31,34^, whereas three studies reported no correlation^21,22,28^ and one study was unable to test for an association^28^. To compare PDO-based drug screen results and clinical response, certain studies chose a clinical parameter which reflects the lesion from which the PDO was obtained rather than the patient’s clinical response, while the latter is most clinically relevant (Table 2 listed as the reference test). In the following sections, we analyse the results in more detail and report pooled results of the clinical validity results.

### Systemic chemotherapy and targeted therapy

#### CRC patients

Four studies reported results regarding the predictive value of PDO drug screen results for treatment response in CRC patients of various disease stages receiving systemic chemotherapy^21,26,28,29^. The TUMOROID and CinClare trials showed that PDO drug screen results were associated with the observed clinical response in patients treated with irinotecan-based regiments^21,26^. In the TUMOROID study, the examined ex vivo drug screen parameters derived from GR were predictive for the best RECIST response to irinotecan-based treatment observed in the lesion from which the PDO was obtained in metastatic CRC (mCRC) patients (*n* = 10 irinotecan and *n* = 12 irinotecan-doublet)^21^. A drug response-based cut-off correctly discriminated between the best RECIST response observed in the lesion from which the PDO was derived in 92% (95% CI 65–99%; calculated using Wilson’s method, 11/12) of patients receiving irinotecan-doublet treatment^21^. Moreover, 50% of patients with the most sensitive in vitro results had significantly longer progression-free survival (PFS, median 169 versus 58 days (digitized from figure), *p* = 0.0278)^21^. In the CinClare trial, ex vivo PDO drug screen results (organoid size) were predictive for clinical response (tumour regression grade upon resection) in 80 locally advanced rectal cancer (LARC) patients receiving neoadjuvant chemoradiation, randomized for capecitabine or capecitabine and irinotecan (CAPIRI), and correctly classified 95% (95% CI 76–91%; 68/80) of the patients^26^.

These results are promising and suggest that PDO drug screen results are predictive for clinical response in CRC patients receiving irinotecan-based treatment. However, there are conflicting results regarding if PDO drug screens are associated with clinical response for oxaliplatin-based treatment. Ganesh et al. ^29^ reported an association in a RC cohort while the TUMOROID trial^21^ and APOLLO trial^28^ did not find an association in mCRC patients. The drug response results (AUC) was significantly associated with the observed PFS in seven RC patients (Ganesh et al. ^29^). This is in contrast to the TUMOROID results in mCRC patients, where none of the ex vivo drug screen parameters were predictive of the RECIST response in the lesion from which the PDO was derived^21^. The APOLLO results support the findings from the TUMOROID study, since the drug response results (AUC) were not different for nine mCRC patients with peritoneal metastases who clinically had response versus no response to 5-fluorouracil and oxaliplatin (FOLFOX) treatment^28^. Interestingly, both Ganesh et al. and the TUMOROID trial reported results for a cohort of patients in which the majority had received oxaliplatin-based treatment prior to deriving the organoids (71% and 60%, respectively)^21,29^. With the reported positive result in the Ganesh et al. study, despite including patients with prior oxaliplatin treatment, it seems that prior treatment does not affect the predictive value of ex vivo PDO drug screens. One aspect which may have influenced the conflicting results between the Ganesh et al. study and the TUMOROID and APOLLO trials is the choice of solvent used to dissolve oxaliplatin for the PDO drug screens, since this is known to affect the activity of oxaliplatin^35^.

#### Upper-gastrointestinal cancer

In a cohort of 15 metastatic gastrointestinal cancer patients treated with different systemic therapies, a binary ex vivo sensitive versus resistant classification of the drug screen results had predictive value, with a 100% sensitivity, 93% specificity, 88% positive predictive value and 100% negative predictive value^11^. Four studies provided descriptive results to compare PDO drug screen results with clinical response in patients with pancreatic cancer^17,23^, non-metastatic gastric cancer (GC)^31^ and non-metastatic oesophageal cancer^27^ patients. The results from the Tiriac et al. and Sharick et al. studies in pancreatic cancer patients receiving chemotherapy regimens suggest that PDOs can function as a predictive biomarker for pancreatic cancer patients. However, the results in gastric and oesophageal cancer patients are preliminary, based on small cohort sizes, and do not yet convincingly show that PDO drug screen results are associated with clinical response in patients. The PDO drug screen results (AUC) of 8/9 (89%, 95% CI 57–98%) pancreatic cancer patients within the Tiriac et al. study, were consistent with clinical outcome (Table 2 with 1–3 patients received a given treatment)^23^. Similarly, in the Sharick et al. study, a cut-off of the OMI index correctly discriminated 7/7 patients based on the clinical recurrence-free survival (RFS)^17^. Patients with a RFS > 12 months had a higher OMI index compared to patients with <12 months RFS^17^. The drug screen of several pancreatic PDOs also contained cancer-associated fibroblasts, for which the in vitro response generally matched the clinical response^17^. Steele et al. reported that in two GC patients receiving epirubicin, oxaliplatin and 5-FU, the clinical response matched the drug response (% cell viability decrease)^31^. Lastly, Li et al. found mixed results in five non-metastatic oesophageal cancer patients receiving one of three combination treatment regimens, with 3/5 having a matching PDO drug screen result with the observed clinical response, while 2/5 patients with a tumour regression grade of 4–5 had limited sensitivity in the PDO drug screens^27^.

#### Other tumour types

Two publications examined the clinical validity of PDO drug screens in patients with ovarian cancer^18,32^. PDO drug response (AUC) was correlated with clinical response in five patients with high-grade serous ovarian cancer who underwent interval debulking surgery for three clinical parameters: histopathological chemotherapy response score, normalization of CA-125 and RECIST response (*p* < 0.01)^32^. No correlation was found with 6 months PFS (*p* = 0.97), however, PDO drug response did correlate with overall survival (<17 months, *p* = 0.0016)^32^. The remaining studies reported descriptive results for 2–3 patients each, shown in Table 2 and Supplementary Table 3, with metastatic ovarian cancer patients receiving carboplatin treatment^18^, metastatic mesothelioma patients receiving cisplatin and pemetrexed^20^, metastatic breast cancer patients receiving tamoxifen^14^ and breast cancer patients receiving neoadjuvant chemotherapy^17^. Considering the small sample size and descriptive nature of the results, we will not elaborate on the results.

#### (Chemo)radiation

In all four studies examining (chemo)radiation, a possible association is reported between PDO drug screens results and clinical response for RC^26,29^, HNSCC^34^ and glioblastoma^30^ patients. The results for the CinClare study were described above, which combined neoadjuvant radiation with capecitabine or CAPIRI^26^, showing a clear association between PDO drug screen results (organoid size) and tumour regression grade upon resection in 80 LARC patients. Ganesh et al. reported that the PDO drug screen AUC was associated with endoscopic clinical response in seven RC patients, based on a descriptive comparison (Table 2)^22^. Similarly, in non-metastatic HNSCC patients, the PDO drug screen AUC descriptively matched clinical response in 86% (95% CI 49–97%; 6/7) of patients receiving postoperative radiotherapy and was inconsistent in 1 patient^34^. A descriptive comparison of the PDO drug screens for radiation and temozolomide treatment (decrease in cell viability) matched the clinical response in five glioblastoma patients, while for two patients no clinical response was reported^30^. Although the results should be validated in larger trials, the results for the predictive value of PDO drug screens in predicting radiation treatment response are promising.

#### Immune checkpoint inhibition

Two studies used co-cultures of PDOs with immune cells to examine the effectivity of immune checkpoint inhibitors (ICI), which require the immune system to orchestrate cytotoxicity^19,22^. The studies showcase the potential of using PDOs in more complex tumour microenvironment co-culture models to predict a variety of treatments. Votanopoulos et al. reported that in 86% (95% CI 49–97%; 6/7) of melanoma patients, immune-enhanced PDO drug screen results (decrease in cell viability) recapitulated the clinical response^19^. In the NICHE trial, the PDO-immune cell co-culture drug screen results (based on the IFN-ƴ production by CD-8+ T-cells) matched the clinical response to neoadjuvant nivolumab and ipilimumab in six CRC patients, all non-responders with proficient mismatch repair (pMMR) tumours^22^. However, the drug screen results were inconsistent in 3/6 patients with clinical response, comprising patients with a pMMR or deficient mismatch repair (dMMR) tumour^22^. Thus, in melanoma patients, immune-enhanced PDO cell viability was associated with treatment response to ICI, indicating that PDO-immune co-cultures drug screen results correlated with treatment response, while in CRC patients receiving neoadjuvant immunotherapy, the IFN-ƴ production by CD-8+ T-cells in co-culture with PDOs did not predict clinical response.

#### Pooled clinical validity results

**Fig. 3:**
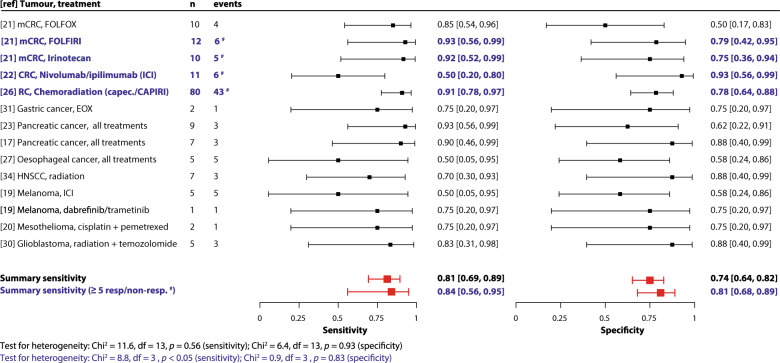
Forest plots of sensitivity and specificity (clinical validity pooled results). A paired forest plot of the sensitivity and specificity of each study and treatment type is shown with 95% confidence intervals. A bivariate meta-analysis was performed to obtain a pooled summary estimate for sensitivity and specificity indicated in the forest plots (1: for all studies that reported results that could be included in this pooled analysis and 2: for studies with ≥5 responders/non-responders). The analysis was performed in R (Version 3.6.1) using the “mada” package^51^. In blue and bold font (^#^) the studies were indicated which were included in the analysis for ≥5 responders/non-responders. Patients who contributed to multiple accuracy estimates: 2 patients received ICI and dabrefinib/trametinib^19^; 3 patients received FOLFOX and irinotecan-based treatment^21^; 3 patients had 2 PDOs each (before and after FOLFOX treatment)^21^ and 1 patient had a synchronous tumour (responder and non-responder)^22^. *Abbreviations:* capec*.* capecitabine, CAPIRI capecitabine + irinotecan, df degrees of freedom, EOX epirubicin + oxaliplatin + 5-FU, FOLFIRI 5-flouruoracil + irinotecan, FOLFOX 5-fluorouracil + oxaliplatin, HNSCC head & neck squamous cell carcinoma, ICI immune checkpoint inhibitors, mCRC metastatic colorectal cancer, RC rectal cancer, ref reference, resp. responder clinically, non-resp*.* non-responder clinically.

To summarize the clinical validity results, we pooled the sensitivity and specificity of PDO-based drug screen results for predicting treatment response. The pooled sensitivity and specificity values for clinical response through PDO-based screening were 0.81 (95% CI 0.69–0.89) and 0.74 (95% CI 0.64–0.82), respectively (Fig. 3 demonstrates the paired forest plots), with a *χ*^2^ test for heterogeneity of 11.6 for sensitivity (*p* = 0.56) and 6.4 for specificity (*p* = 0.93). Considering the small sample sizes, we repeated the meta-analysis for studies with ≥5 responder and non-responder patients^21,22,26^ and obtained similar results (pooled sensitivity 0.84 (95% CI 0.56–0.95) and specificity 0.81 (95% CI 0.68–0.89)), with a *χ*^2^ test of 8.8 for sensitivity (*p* < 0.05) and 0.9 for specificity (*p* = 0.83). The pooled sensitivity and specificity values are likely an overestimation, since studies that did not report quantitative results necessary for the meta-analysis could not be included and since not all studies used a pre-defined index test. As such, we cannot exclude publication or outcome reporting bias in the results. The area under the receiver operator curve (AUROC) for discriminating clinical response using various index tests clinical validity results is summarized in a forest plot in Supplementary Fig. 1. The pooled results are an indication of the overall performance of PDOs in predicting response across different tumour types and treatments for the available evidence. Despite heterogeneity in tumour type, treatment and end points used in the studies, we do not see heterogeneity in our data. However, the pooled results should be interpreted cautiously considering that PDOs may predict differently between tumour types and treatments. Future studies will enable meta-analysis per tumour and treatment type; due to limited evidence this is currently not possible.

#### Effect of spatial intrapatient heterogeneity on clinical validity

For PDO-based drug screen results to be clinically valid in patients with advanced cancer, PDOs should be able to act as a predictive biomarker for treatment response in the patient as a whole, without being limited by intratumoral heterogeneity^36^. Although PDOs are heterogenous^9,17^, a PDO is derived from a single biopsy or surgical resection, representing a snapshot of one spatial lesion. Intra-patient heterogeneous PDO drug screen responses were relatively uncommon in a cohort of CRC patients, with pharmacological profiles of PDOs obtained from multiple CRC liver metastases in 10 patients largely clustering together patient-wise and inter-metastatic heterogeneity being observed in <1/10th of all drug–patient comparisons^37^. Interestingly, Sharick et al. were able to assess the heterogeneity on a cellular level of PDOs during treatment using OMI^17^. The PDOs of pancreatic cancer patients with a RFS > 12 months had a lower degree of metabolic heterogeneity during treatment (versus control PDOs), while PDOs from patients with a RFS < 12 months had increased heterogeneity in treatment PDOs compared to control PDOs^17^.

Five studies reported spatial intrapatient heterogeneity in drug responses in PDOs derived from distinct cancer lesions per patient, although the clinical implications are unclear^11,21,23,28,32^. Vlachogiannis et al. demonstrated that the mixed clinical response seen in a mCRC patient to trifluridine/tipiracil (TAS-102) treatment, with PD in one liver metastasis and stable disease (SD) in a second liver metastasis, was reflected in the PDO drug screen results, with an eightfold difference in GI_50_ between the PDO derived from the sensitive metastasis and the PDOs derived from the PD metastasis^11^. In seven ovarian cancer patients (2–4 PDOs/patient), de Witte et al. demonstrated that all related PDOs exhibited a differential drug response to at least one drug, and that the differential drug response could only be partially linked to genetic heterogeneity^32^. Based on a small subset of patients with paired PDOs from different tumour regions, the remaining studies demonstrated heterogeneity in drug response, without correlating this to the response seen in the patient. These studies show that the effect is treatment^21,23^ and patient specific^28^.

The studies which reported a correlation or predictive value for PDO individualized tumour response testing and treatment response are based on results using the response in the lesion from which the PDO was obtained^21,26^ and analysing the patient’s response as a whole, e.g. RECIST response^11,29,32^. The TUMOROID study, which primarily examined the clinical response in the lesion from which the PDO was obtained, also showed that patients with the 50% most sensitive drug screen results had a significantly longer PFS for 5-FU + irinotecan combination treatment^21^. Although intrapatient heterogeneity in PDO drug screen results has been shown, the current available results indicate that PDOs are able to act as a predictive biomarker for the patient’s treatment response as a whole, and thus are not significantly impeded by intrapatient heterogeneity. However, these results should be confirmed in future studies.

## Clinical utility: feasibility of using PDO drug screens to predict treatment response

If PDOs are to be effectively translated to the clinic for precision medicine, their clinical utility must be proven. Three feasibility aspects are important for the clinical utility of PDO-based individualized tumour response testing: (1) having a sufficiently high PDO establishment rate to balance the burden incurred through diagnostic interventions to obtain tissue to culture PDOs; (2) avoiding unnecessary treatment delay by minimalizing the time from obtaining tissue for culturing PDOs to analysis of PDO drug screen results and (3) the use of PDO-guided treatment should be beneficial for patients through increased survival and/or quality of life by either using PDOs to select patients for the most optimal standard of care treatment option or through identifying novel treatment candidates. The clinical benefit of PDO-guided treatment compared to (standard of care) physician-guided treatment has not been assessed. However, the APOLLO trial is the first to provide PDO-guided treatment to patients. We discuss all three feasibility aspects below.

The organoid establishment rate was reported in 12 studies, ranging from 31% to 90%. We performed a random effects pooled analysis of the reported organoid establishment rates per study using a generalized linear mixed model. Two analyses were performed: sample-level (the proportion of established organoids per total number of samples obtained) and patient-level (the proportion of patients with established organoids per total number of patients sampled), including all reported establishment rates (except when only an approximation was reported^34^). The pooled organoid establishment rate was 68.5% (95% CI 56.5–78.5%; *I*^2^ = 89%) in seven studies reporting sample-level organoid establishment rates^11,14,21,23,26,27,29^ and 68.0% (95% CI 54.9–78.8%; *I*^2^ = 83%) in eight studies reporting patient-level organoid establishment rates^17,19,21,23,27–30^ (Supplementary Fig. 2 demonstrates the forest plots). An establishment rate of ~70% may be high enough to balance potential burdens for patient in obtaining tissue for culturing PDOs and may improve through developments in establishment techniques. The highest establishment rates were observed in melanoma (resections: 90%^19^) and rectal cancer patients (77–86%^26,29^). Only tumour biopsy cellularity was found to be associated to PDO establishment rate^11^, while site of tissue sampling (primary tumour versus metastasis) and prior treatment were not found to be different in patients with or without established PDOs^17,21^.

The acceptable time from tissue sampling to obtaining drug screen results, without delaying treatment will vary depending on the clinical situation. Unfortunately, only two studies reported the time needed from tissue sampling to obtaining drug screen results (<8 weeks for all patients^28^ and <20 days in a pilot for 1 patient^32^). The time required to establish PDOs after obtaining tissue can vary greatly, as exemplified in a study for pancreatic cancer resections (median 10 days, inter-quartile range, IQR 6–12) versus breast cancer biopsies (median 34 days, IQR 27–51)^17^. The period from obtaining tissue to drug screen results may be reduced by minimizing the number of PDOs needed and duration of drug exposure while maintaining analytical validity.

The APOLLO trial shows promise that PDO-guided treatment is feasible and may offer additional treatment options for treatment refractory patients^28^. Patients with peritoneal mCRC and disease progression despite standard systemic treatment were screened using an adapted CRC-focused panel of clinically available treatments and two patients received PDO-guided off-label treatment (described in Supplementary Table 3). Although this study illustrates the feasibility of performing organoid-based treatment stratification, considering the small number of patients no firm conclusion can be drawn concerning the clinical utility of PDO-guided treatment. However, the study does highlight the potential for PDO drug screens to identify novel treatment candidates for patients which otherwise would not have been available. PDO drug screens can be performed in a high throughput manner, enabling rapid screening of large libraries of therapeutic agents to identify new agents or new combinations of agents for a patient or subgroup of patients^38^. Effective anti-cancer treatments are often combination regimens and thus libraries of single targeted agents may not result in identification of clinically effective agents. In conclusion, demonstrating the clinical utility of PDOs requires demonstrating that patients benefit through increased survival or quality of life, which can potentially be achieved by either using PDOs to identify which standard of care drugs are most effective (avoiding exposure to ineffective drugs and their associated toxicity) or through identifying new therapy candidates through library screening of non-standard of care drugs.

## Recommendations for future studies

Several recommendations for future research can help accelerate the implementation of PDO drug screens as a predictive biomarker in the clinic. We will first address recommendations for methodology and reporting of studies. Secondly, new innovations in PDO drug screens are examined which can improve the reproducibility and automation of drug screens. And lastly, we will describe aims of future studies to accelerate the transition of PDOs to the clinic.

### Standardized methodology and reporting

Researchers should aim to adhere to methodological standards when reporting results, to facilitate study quality assessment (including potential biases) and study result interpretation^39^. The REporting recommendations for tumour MARKer prognostic studies (REMARK) guidelines can be used to standardize reporting for studies examining PDOs as a predictive biomarker in patients with cancer^39^. We wish to highlight several related aspects applicable to the methodology and reporting for future studies, which are specific for PDO predictive biomarker studies.

Given the heterogeneity in PDO drug screen set-up used, a validated, standardized experimental design may offer benefits. It allows researchers to avoid unnecessary time in validating a new experimental design, to use previously tested organoid lines to validate results and to prospectively validate published PDO-based diagnostic tests. This may be achievable, since the APOLLO trial demonstrated that using a similar experimental design in two laboratories resulted in significantly correlated results (Pearson’s *r* = 0.96, *p* < 0.05)^28^. Furthermore, physicians could use a database of published drug screen results to assess if a patient’s drug screen is relatively resistant or sensitive.

To start with, PDO culturing and screens should aim to use materials which are not animal-derived or serum-based. The majority of organoid studies used animal-derived extracellular matrices (e.g. Matrigel^®^), which are biologically variable and contain animal-derived growth factors^40^. Animal-derived matrices can theoretically reduce the reproducibility of drug screens and influence PDOs in culture, while also reducing the extent to which the model reflects the physiological setting. New synthetic hydrogels, which are fully defined and growth factor-free, have proven to support the establishment of human PDOs from single cell suspensions and are amenable to drug screens^41–44^. Future studies should explore if synthetic hydrogels can be used to establish PDOs from human tissue and whether the use of synthetic hydrogels improves the reproducibility of drug screens. Similarly, serum-free Wnt growth factor supplements are increasingly available, enabling organoid culture medium to become serum-free^16^.

Subsequently, one important aspect is transparent reporting of the chosen definition for clinical response and in vitro response, to ensure reproducible study results and interpretation for clinical applicability. In the methods, it should be clear if the chosen end points were part of a pre-defined statistical analysis. As well, drug screens of combination treatments should analyse response of the combination treatment by adding both agents directly in vitro, rather than analysing the response separately, to best model the treatment given to the patient.

Finally, as mentioned previously, detailed reporting of the establishment rate, including how successful establishment of PDOs was defined and quality control to verify that PDOs represent the original tumour, and time needed to obtain PDO drug screen results from obtaining tissue will help in validating the feasibility of using PDOs as a biomarker. Reporting results of feasibility aspects for PDO establishment, including the location and type of tissue obtained, establishment rate per patient and per sample obtained, and features (e.g. patient demographics, molecular status, etc.) found to be associated with successful establishment of PDOs, will aid researchers in improving PDO establishment techniques. If PDOs derived from primary disease (or earlier treatment lines) have predictive value for patients with metastatic disease or later treatment lines, which is currently unknown, the time to obtaining results can be minimized by culturing PDOs early in the course of the disease.

### Innovations in PDO drug screens

Organoid-based drug screens are developing rapidly, offering new techniques and materials which can improve the reproducibility, high throughput design and automation of PDO screening. A newly developed automated microfluidic platform for PDO drug screening enables the addition of drugs at different time points, allowing drug screens to more closely resemble combination treatment regimens given to patients^45^. Such automated platforms may be compatible with image-based analysis^46^, which in contrast to single end points such as cell viability, allows researchers to assess multiple end points, better resembling the full drug response in PDOs. Furthermore, PDO drug screens are being optimized to become more high throughput^47^. These developments will aid in automating PDO drug screens, decreasing the amount of PDOs needed and developing read-outs which more accurately represent the true drug response in PDOs compared to traditional read-outs.

### Aims for future studies

The clinical validity of using PDOs to predict treatment response should be confirmed in studies with a larger group of patients, ideally in a specific clinical setting for one tumour and treatment type. The desired predictive test qualities may vary for a given clinical setting, e.g. the amount of treatment options still available and the a priori chance that a patient will respond to a given treatment. The predictive value of PDOs may be tumour or treatment specific, given the conflicting results regarding oxaliplatin-containing treatment within different mCRC studies and ICI treatment for melanoma and CRC. Having results available for specific subgroups will give us further knowledge concerning the settings in which PDOs may offer predictive value for patients.

Subsequently, the use of more complex PDO models, such as co-cultures may be necessary to accurately predict treatment response for certain treatments where the tumour microenvironment—including the immune system—affects treatment sensitivity (e.g. immunotherapy)^19,22,48^. The available evidence suggests that co-cultures may not be necessary to predict treatment response for chemotherapeutics, since the discussed studies on mono-culture PDO models could predict chemotherapy response. However, more complex drug screen models may increase the predictive ability for treatments, with increasing anti-cancer agents targeting the tumour microenvironment as well as the tumour itself and the possibility to include effects of drug metabolism.

Ultimately, the clinical value of using PDO individualized tumour response testing should be proven by comparing clinical outcomes, such as progression-free survival or response rates, in randomized clinical trials comparing physician guided standard of care treatment versus assay-guided treatment derived from PDO drug screens^49^. Patients receiving assay-guided treatment should benefit clinically and ideally this benefit should be cost effective compared to standard of care treatment or, for example, genomic-guided treatment^49,50^.

## Conclusions

The currently available results offer an optimistic perspective that individualized tumour response testing using PDOs have clinical validity as a predictive biomarker for cancer patients. The pooled sensitivity and specificity for discriminating patients with a clinical response through PDO-based screening were 0.81 (95% CI 0.69–0.89) and 0.74 (95% CI 0.64–0.82), respectively, although this is an estimation since not all studies reported results which could be used for the pooled analysis and not all studies used a pre-defined index test when analysing results. The pooled results are an indication of the overall performance of PDOs in predicting response across different tumour types and treatments for the available evidence. However, they should be interpreted cautiously considering that PDOs may predict differently between tumour types and treatments. The current evidence is strongest for CRC patients, with larger studies showing a correlation between PDO-based drug screen results and systemic therapy/radiation treatment response and with smaller studies showing promising descriptive results for other tumour and treatment types. Associations were found for a broad variety of tumours, treatment types and encompassing several drug screen parameters, albeit, not consistently for all tumour types and treatments. Prior to being able to implement PDO-based drug screens in the clinic, the results should be validated in similar, larger patient cohorts. The current challenge is to prove that PDO-based individualized tumour response testing is feasible, by optimizing organoid establishment rates and time to obtaining PDO-based screening results. The results regarding clinical validity of PDOs as a predictive biomarker are promising and ultimately the clinical utility should be proven by demonstrating that PDO-based individualized tumour response testing is cost effective and offers clinical benefit for patients. If PDOs can be established for the majority of patients within a feasible time frame, this potential predictive biomarker can facilitate personalized medicine for a group of patients for whom there is a great need for valid predictive biomarkers.

### Reporting summary

Further information on research design is available in the Nature Research Reporting Summary linked to this article.

## Supplementary information

Supplementary Information

Reporting Summary

## Data Availability

All aggregated data used in the analysis is reported in the manuscript and/or supplementary materials. The aggregated datasets analysed during the current study are available from the corresponding author on reasonable request.

## Code availability

All analysis was performed in R (Version 3.6.1) using publicly available packages^51^. The packages used for analysis are indicated in the legend of each corresponding figure. The used code scripts are available from the corresponding author on reasonable request.

## Materials availability

Figure 1 was designed using resources from Flaticon.com.
