# Supplementary material for: Patient-derived organoids as a predictive biomarker for treatment response in cancer patients
Source: NPJ Precis Oncol. 2021 Apr 12;5:30. doi: 10.1038/s41698-021-00168-1 (PMC8042051; doi:10.1038/s41698-021-00168-1)
Supplement: Supplementary file 1 — Supplementary Information [file 41698_2021_168_MOESM1_ESM.doc]

**Supplementary Materials**

Supplementary Table 1. Literature search strategy

Supplementary Table 2. Drug screen experimental designs

Supplementary Table 3. Clinical association with drug screen - descriptive results

Supplementary Figure I. Forest plot of reported AUROC per study, treatment and index test

Supplementary Figure II. Meta-analysis and forest plots of reported organoid establishment rates per study

Supplementary Table 4. Reported organoid establishment rates

Supplementary Table 1. Literature search strategy

| **Search terms & databases used** | **Pubmed:**  (Organoid*[tiab] OR Tumoroid*[tiab] OR PDO[tiab] OR "patient-derived organoid"[tiab]) AND (predict*[tiab] OR correlate*[tiab] OR personalized[tiab] OR precision[tiab]) AND (cancer[tiab] OR neoplasm[tiab] OR tumor[tiab]) NOT review[tiab]  No filters.  **Embase:**  (organoid*:ab,ti OR tumoroid*:ab,ti OR pdo:ab,ti OR 'patient-derived organoid':ab,ti) AND (predict*:ab,ti OR correlate*:ab,ti OR personalized:ab,ti OR precision:ab,ti) AND (cancer:ab,ti OR neoplasm:ab,ti OR tumor:ab,ti) NOT review:ab,ti  Filters: source from article, article in press, data papers, letter or short survey. |
| --- | --- |
| **Inclusion criteria** | Original research articles, reporting results on:  **Population:** patients with cancer, with clinical response data available for a given treatment.  **Intervention:** organoids established using tissue obtained from the tumour (without xenograft models in the establishment process), on which drug screens were performed  **Control:** clinical response of the patient to a given treatment  **Outcome:** compare the *in vitro* organoid drug screen response with the clinical response of the patient to a given treatment  >1 patient results comparing the PDO-based drug screen result and clinical outcome must be reported. |
| **Results search** | Pubmed: 385 results  Embase: 286 results  **Total: 671 results**    Remove duplicates: -268 results  **Total: 403 results**  Screen title/abstract: -374 results  **Total: 29 results**  Screen full-text article: -13 results (all excluded due to not reporting comparison with patient-level response for a given treatment)  **Identified studies for systematic review: 16**  + 1 results (from cross-referencing / citations of included studies)  **Included studies in systematic review: 17.** |

***Abbreviations:*** title abstract (tiab or ab,ti).

**Supplementary Table 2. Drug screen experimental designs & authentication of tumour organoids**

| **Study**  **(Author, year)** | **Quality control of tumour organoids** | **Drug screen model** | **Timeline drug screen** | **Drug exposure duration (days)** | **End point of drug screen** | **Blinding of drug screen and/or clinical parameters** |
| --- | --- | --- | --- | --- | --- | --- |
| Ooft, 2019 *(21)* | DNA sequencing (discard if identity scores <0.9). | Embedded in Geltrex (1:2 ratio of medium:matrix). | Day 1: 4-day old PDOs; baseline read-out; drug exposure.  Day 6: end-point. | 6 | Cell viability (CellTiter-Glo 3D).  Calculate growth rate inhibition metrics (GR). | Drug screens were performed by 2 researchers (2^nd^ was blinded for clinical parameters). No other blinding reported. |
| Chalabi, 2020 *(22)* | DNA sequencing (discard if identity scores <0.9). | PDO lymphocyte co-culture (patient-derived TIL’s) in suspension. | Day 0: 1 day prior stimulate PDOs with IFN- ƴ.  Day 1: single cell PDOs + PBL’s (1:20 ratio), co-stimulate with IL-2 + anti-CD-28 (plate-bound); drug exposure.  Every 2-3 days: refresh medium (IL-2 + drugs).  Day 14: end-point. | 14 | Quantification of IFN-ƴ production by CD8+ T-cells. | No blinding performed. |
| Ganesh, 2019 *(29)* | Histopathology confirmation of morphology.  DNA sequencing. | Embedded in Matrigel. | *Drug screen:*  Day 1: 2-3 day old PDOs; drug exposure.  Day 3: refresh medium with drugs.  Day 6: end-point.  *Radiation:*  Day 1: irradiate PDOs.  Day 8-13: end-point. | Chemotherapy: 6  Radiation:  8-13 | Cell viability (CellTiter-Glo). | Investigators performing radiation on PDOs were blinded for patient characteristics. No other blinding reported. |
| Yao, 2020  *(26)* | Histopathology confirmation of morphology.  DNA sequencing. | Embedded in Matrigel. | *Drug screen:*  Day 1: PDOs (100 uM diameter) drug exposure.  Day 3: refresh medium with drugs.  Every subsequent 3 days: refresh medium.  Day 24: end-point.  *Irradiation:*  Day 1: PDOs (100 uM diameter) irradiated.  Every 3 days: refresh medium.  Day 24: end-point. | 24 | PDO size (day 24 versus 0, imaging). | No blinding reported. |
| Narasimhan, 2020 *(28)* | Histopathology confirmation of morphology.  DNA sequencing.  Xenograft to confirm tumorigenicity. | Suspension (5% Matrigel). | Day 1: PDOs; drug exposure.Day 6: end -point. | 6 | Cell viability (CellTiter-Glo 2D). | No blinding reported. |
| Vlachogiannis, 2018 *(11)* | Histopathology confirmation of morphology.  DNA sequencing. | Embedded in Matrigel. | Day 1: 3-day old PDOs; drug exposure.  Every 2 days: refresh medium with drugs.  Day 9-11 (growth rate-dependent): end-point. | 9-11 | Cell viability (CellTiter-Glo). | Researchers were blinded to the patients’ response. |
| Steele, 2019 *(31)* | Niche-dependency.  RNA sequencing.  Xenograft to confirm tumorigenicity. | Unclear. | *Monotherapy:*  Day 1: drug exposure.  Day 2 (48 hrs): end-point.  *Combination (used for correlation):*  Day 1: drug exposure (use IC_50_ per PDO).  Every 24 hrs: flow cytometry of single cells. | Monotherapy: 2  Combination: 4 | *Monotherapy:* MTS assay.  *Combination:* cell viability via flow cytometry. | No blinding reported. |
| Tiriac, 2018 *(23)* | DNA sequencing (discard if does not have any known pathogenic mutations). | Suspension (10% Matrigel) | Day 1: 1-day old PDOs; drug exposure.  Day 5: end-point. | 5 | Cell viability (CellTiter-Glo). | No blinding reported. |
| Sharick, 2020 *(17)* | Xenograft to confirm tumorigenicity. | Embedded in Matrigel. | Day 1: drug exposure.  Day 2: remove gemcitabine, paclitaxel, SN-38 and oxaliplatin (not other drugs).  Day 3: end-point. | 3 (some cases 5 or 7) | Optical metabolic imaging (OMI) index, which reflects the metabolic state of cells (relative to average control values). | No blinding reported. |
| Li, 2018 *(27)* | Niche dependency.  Histopathology confirmation of morphology.  DNA & RNA sequencing. | Suspension (25% BME-2). | Day 1: plate PDO suspension.  Day 2: drug exposure.  Day 7: end-point. | 6 | Cell viability (CellTiter-Glo). | No blinding reported. |
| Driehuis, 2019 *(34)* | Niche-dependency.  Histopathology confirmation of morphology.  DNA & RNA sequencing.  Xenograft to confirm tumorigenicity. | *Drug screen:*  Suspension (5% BME).  *Radiation:*  Embedded in BME. | *Drug screen:*  Day 1: 2-day old PDOs; drug exposure.  Day 5: end-point.  *Radiation:*  Day 1: 2-day old PDOs; irradiated in water bath, medium change.  Day 4: end-point. | Drug screen: 5  Radiation: 4 | Cell viability (CellTiter-Glo 3D). | No blinding reported. |
| Sachs, 2018 *(14)* | Histopathology confirmation of morphology.  DNA (exclude from analysis if cross-contamination seen) & RNA sequencing. | Suspension (5% BME), bottom layer BME. | Day 1: 5-7 day old PDOs; drug exposure.  Day 5: end-point. | 5 | Cell viability (CellTiter-Glo 3D). | No blinding reported. |
| Phan, 2019 *(18)* | Histopathology confirmation of morphology.  Xenograft to confirm tumorigenicity. | Embedded in Matrigel, plated as mini-ring. | Day 1: 2-day old PDOs; drug exposure.  Day 2,3: refresh medium with drugs.  Day 4: end-point. | 3 | PDO size and count (brightfield imaging).  Cell viability (CellTiter-Glo 3D). | No blinding reported. |
| De Witte, 2020 *(32)* | Histopathology confirmation of morphology.  DNA sequencing. | Suspension (2% BME), bottom layer BME. | Day 1: PDOs; drug exposure.  Day 5: end-point. | 5 | Cell viability (CellTiter-Glo 2D). | Pathological assessment was blinded. No other blinding reported. |
| Votanopoulos, 2019 *(19)* | Histopathology confirmation of morphology. | Embedded in ECM-mimicking HA/collagen-based hydrogel.  Immune-enhanced PDOs OR PDOs + immune cells (1:1 ratio). | Day 1: 7-day old PDOs (from establishment); drug exposure.  Day 3 (72h): end-point. | 3 | Cell viability (CellTiter-Glo 3D).  Live/Dead staining (calcein-AM and ethidium) and confocal imaging. | Partial blinding: Clinical information  was not shared with the laboratory, with the exception of  type of tumor and type of prior treatments. |
| Mazzocchi, 2018 *(20)* | Histopathology confirmation of morphology. | Embedded in HA + gelatin hydrogel in tumour-on-chip microfluidic device. | Day 1 (growth dependent): drug exposure.  Day 7 or 14: end-point. | 7 or 14 | Live/Dead staining (calcein-AM and ethidium) via immunofluorescence. | No blinding reported. |
| Jacob, 2020 *(30)* | Histopathology confirmation of morphology.  DNA & RNA sequencing.  Xenograft to confirm tumorigenicity. | Suspension. | Day 1: irradiation + temozomide exposure.  Day 3,5: refresh medium.  Day 7: end-point. | 7 | Cell viability staining (Ki67 +DAPI) via immunofluorescence. | No blinding reported. |

*Abbreviations:* BME (basement membrane extract), CD8+ T-cells (cytotoxic T-cells), CD-28 (cluster of differentiation 28), ECM (extracellular matrix), DNA (deoxyribonucleic acid), Gy (gray), HA (hyaluronic acid), IL-2 (interleukin-2), IFN-ƴ (interferon-gamma), kV (kilovolt), mA (milliampere), MTS (3-(4,5-dimethylthiazol-2-yl)-5-(3-carboxymethoxyphenyl)-2-(4-sulfophenyl)-2H-tetrazolium)), PBL’s (peripheral blood lymphocytes), PDOs (patient-derived organoids), RNA (ribonucleic acid), TIL’s (tumor infiltrating lymphocytes).

**Supplementary Table 3. Clinical association with drug screen - descriptive results**

| **Tumour type**  **[Publication]** | **Treatment** | **# patients/ treatment** | | **# events**  **(clinical non-responders)** | **PDO drug screens & clinical response parameters associated** | | **Descriptive results** |
| --- | --- | --- | --- | --- | --- | --- | --- |
|  |  | **Patients** | **PDOs** |  | **Index test**  **(PDO)** | **Reference test (Clinic)** |  |
| mCRC [Narasimhan *(28)*] | FOLFIRI | 4 | 4 | 4 | AUC. | PD versus PR/SD/CR. | Not possible to compare since all patients had PD. |
|  | Regorafenib | 1 | 1 | 1 | AUC. | PD versus PR/SD/CR. | AUC resistant & patient failed to respond. |
|  | Vandetanib **(PDO-guided treatment)** | 1 | 1 | 1 | AUC. | PD versus PR/SD/CR. | AUC with response, but no clinical response (discordant). |
|  | Gemcitabine  **(PDO-guided treatment)** | 1 | 1 | 0 | AUC. | PD versus PR/SD/CR. | AUC sensitive & patient had PR to gemcitabine-capecitabine (3 month evaluation). |
| mGIC [Vlachogiannis *(11)*] | TAS-102 (mCRC) | 4 | 6 | 2 | Not specified. | Not specified. | 1 patient with mixed response had 8-fold difference in GI_50_ between SD versus PD metastasis-derived PDO. In 3 other patients (2 with SD, 1 resistant), PDO response matched clinical response. |
|  | Cetuximab (mCRC) | 4 | 4 | 3 | Not specified. | Not specified. | In 2 primary resistant patients & 1 PD patient, PDOs showed no response. In 1 patient with slow growing metastasis, PDO had marginal response. |
|  | Paclitaxel (mGC) | 3 | 4 | 3 | Not specified. | Not specified. | In patient with 2 PDOs (prior and after PD), the PDO obtained prior to PD had GI50 which was ¼ of GI50 of PDO obtained after PD and which matched GI50 of 2 other patients with PD. |
|  | 5-FU + cisplatin (mGOC) | 2 | 2 | 1 | Not specified. | Not specified. | In a chemosensitive versus refractory patient, GI_50_ differed by 10-fold. |
| Breast cancer  [Sharick *(17)*] | AC-T (breast cancer, neoadjuvant). | 3 | 3 | 1 | OMI index | Pathological: RCB | The patient with clinical RCB III (extensive residual disease) had the lowest OMI index (Glass ∆ 1.97) compared to the patients with clinical RCB I/II (minimal/ moderate disease) with OMI index of 3.76 and 6.23, respectively. The OMI index decreased upon AC-T exposure in the PDOs (Glass’s ∆ <1.9, *p*<0.0001), while the OMI index heterogeneity altered to varying degrees. |
| Breast cancer [Sachs *(14)*] | Tamoxsifen. | 2 | 2 | 1 | Not specified. | Not specified. | Location of DRC (relative to others) matched clinical response. |

**Legend:** Results concerning the association or predictive value between PDO drug screens and clinical response are displayed for studies reporting only descriptive results, along with the publication, tumour type & stage, parameters compared, treatment and number of patients per treatment. *Abbreviations:* 5-FU (5-fluorouracil), AC-T (doxorubicin + cyclophosphamide + paclitaxel), CAPIRI (capecitabine + irinotecan), AUC (area under the curve), CR (complete response), FOLFIRI (5-FU + irinotecan), GI_50_ (concentration that inhibits growth of cancer cells by 50%), mCRC (metastatic colorectal cancer), mGC (metastatic gastric cancer), mGIC (metastatic gastrointestinal cancer), mGOC (metastatic gastroesophageal cancer), OMI (optical metabolic imaging), PD (progressive disease), PDO (patient-derived organoid), PR (partial response), RCB (residual cancer burden grade), SD (stable disease), TAS-102 (trifluridine/tipiracil).

**Supplementary Figure I. Forest plot of reported AUROC per study, treatment and index test**


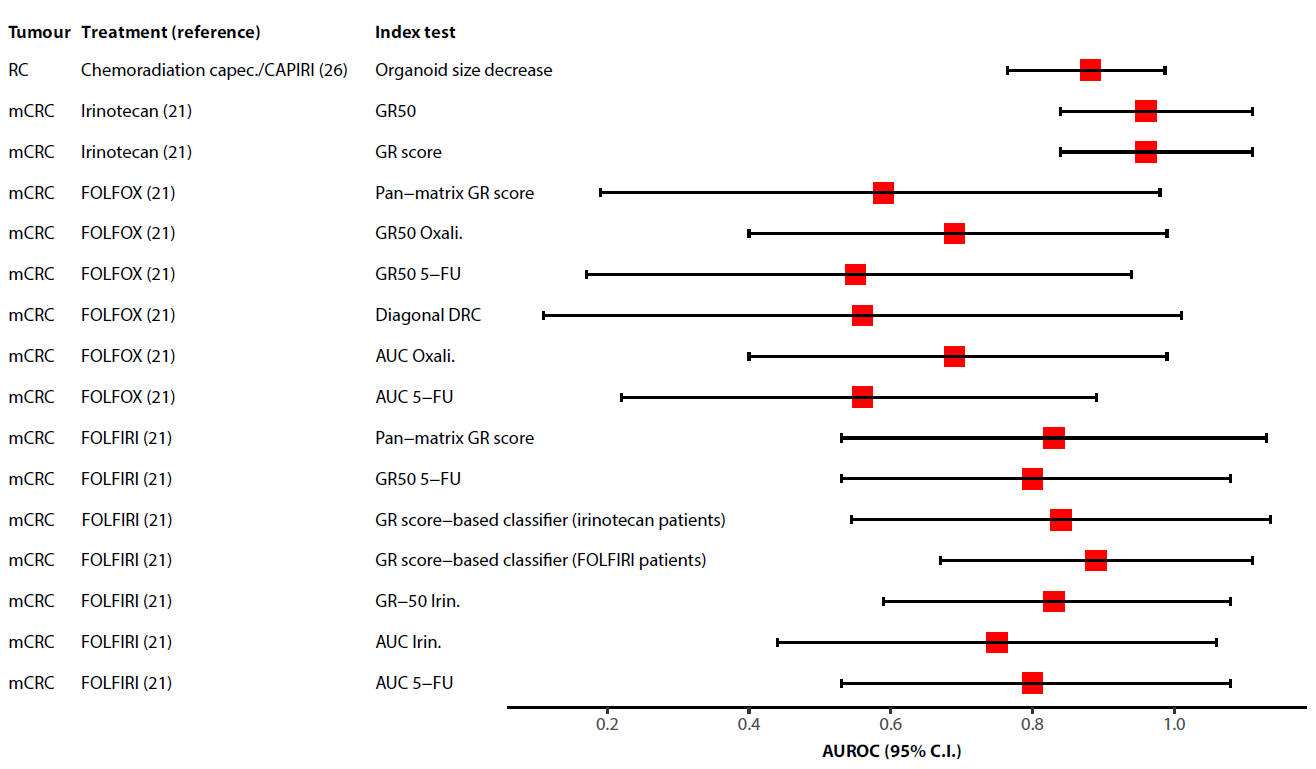


**Legend*:*** A forest plot for the reported AUROC per study (with 95% C.I.), treatment and index test (results were digitized from the figures from Ooft *et al.*). *Abbreviations:* 5-FU (5-fluorouracil), AUROC (area under the receiver operator curve), capec. (capecitabine), CAPIRI (capecitabine + irinotecan), C.I. (confidence interval), DRC (drug response curve), FOLFIRI (5-fluorouracil + irinotecan), FOLFOX (5-fluorouracil + oxaliplatin), GR (growth rate metrics), GR50 (50% GR), irin. (irinotecan), mCRC (metastatic colorectal cancer), oxali. (oxaliplatin), RC (rectal cancer). The analysis was performed in R (Version 3.6.1) using the “mada” package ^51^.

**Supplementary Figure II. Meta-analysis and forest plots of reported organoid establishment rates per study**

**Legend:** A random effects meta-analysis of the reported organoid establishment rates per study (listed in Supplementary Table 4) was performed using a generalized linear mixed model. **a)** Forest plot for the proportion of established organoids per total number of samples obtained (illustrated by the red square, with 95% C.I. error bars); **b)** Forest plot for the proportion of patients with established organoids per total number of patients sampled (illustrated by the red square, with 95% C.I. error bars). *Abbreviations:* C.I. (confidence interval), mBC (metastatic breast cancer), mCRC (metastatic colorectal cancer), mGC (metastatic gastric cancer), mGOC (metastatic gastroesophageal cancer), RC (rectal cancer). ^+^Specimens were also obtained via rapid autopsy procedure. *Operative specimens at staging laparoscopy, cytoreductive reductive surgery and HIPEC, or percutaneous biopsies. The establishment rates for Sharick *et al.* (2020) differ from the published rates, due to the study including patients with pancreatic intraepithelial neoplasia (*n=2*), which were excluded from this analysis. The analysis was performed in R (Version 3.6.1) using the “binom”, “tidyverse”, “metafor” and “meta” packages ^51^.

**Supplementary Table 4. Reported organoid establishment rates**

| **Study (author, year)** | **No. samples with established organoids** | **No. samples for organoid culture** | **Sample-level establishment rate (%)** | **No. patients with established organoids** | **No. patients with samples for organoid culture** | **Patient-level establishment rate (%)** | **Tumour type** | **Tissue** | **Comments** |
| --- | --- | --- | --- | --- | --- | --- | --- | --- | --- |
| Ooft, 2019 | 40 | 63 | 0,63 | 34 | 57 | 0,60 | mCRC | Biopsies. |  |
| Ganesh, 2019 | 65 | 84 | 0,77 | 41 | 58 | 0,71 | RC | Resections / biopsies. |  |
| Yao, 2020 | 96 | 112 | 0,86 |  |  |  | RC | Biopsies. |  |
| Narasimhan, 2020 |  |  |  | 19 | 28 | 0,68 | mCRC | Resections / biopsies (via CRS/HIPEC). |  |
| Vlachogiannis, 2018 | 77 | 110 | 0,70 |  | 71 |  | mCRC, mGC, mGOC | Biopsies. |  |
| Tiriac, 2018 | 114 | 152 | 0,75 | 101 | 138 | 0,73 | pancreatic cancer | Resections / biopsies (also included rapid autopsies). | 75% (all organoid attempts, 72% fine-needle biopsies to 78% resection). |
| Sharick, 2020 |  |  |  | 12 | 20 | 0,60 | pancreatic cancer | Resections. | Differs from publication. Published rates included 2x pancreatic intraepithelial neoplasia (are not malignant). |
| Li, 2018 | 10 | 32 | 0,31 | 10 | 32 | 0,31 | esophageal cancer | Esophagectomy. |  |
| Driehuis, 2019 |  |  |  |  |  |  | HNSCC | Resections / biopsies. | ~ 60%, no further data available. |
| Sharick, 2020 |  |  |  | 13 | 24 | 0,54 | breast cancer | Resections. |  |
| Sachs, 2018 | 95 | 155 | 0,61 |  |  |  | mBC | Lumpectomy. | Through improvements, latest rate >80%. |
| Votanopoulos, 2019 |  |  |  | 9 | 10 | 0,90 | melanoma | Resections. |  |
| Jacob, 2020 |  |  |  | 53 | 58 | 0,91 | glioblastoma | Lumpectomy. |  |

**Legend:** The organoid establishment rates (per samples obtained for organoid culture and per patient with samples available for organoid culture) were extracted from the original studies. For studies not listed in this table, an organoid establishment rate was not reported. *Abbreviations:* HNSCC (head & neck squamous cell carcinoma), mBC (metastatic breast cancer), mCRC (metastatic colorectal cancer), mGC (metastatic gastric cancer), mGOC (metastatic gastric-esophogeal cancer), no. (number) and RC (rectal cancer).
